# Vitamin D Receptor Regulates the Expression of the Grainyhead-Like 1 Gene

**DOI:** 10.3390/ijms25147913

**Published:** 2024-07-19

**Authors:** Agnieszka Taracha-Wisniewska, Emma G. C. Parks, Michal Miller, Lidia Lipinska-Zubrycka, Sebastian Dworkin, Tomasz Wilanowski

**Affiliations:** 1 Faculty of Biology, Institute of Genetics and Biotechnology, University of Warsaw, 02-096 Warsaw, Poland; a.taracha-wisniewska@uw.edu.pl (A.T.-W.); l.lipinska-zubrycka@uw.edu.pl (L.L.-Z.); 2 Department of Physiology, Anatomy and Microbiology, La Trobe University, Melbourne, VIC 3086, Australia; e.parks@latrobe.edu.au (E.G.C.P.); s.dworkin@latrobe.edu.au (S.D.); 3 Nencki Institute of Experimental Biology of Polish Academy of Sciences, 02-093 Warsaw, Poland; miller.michal.89@gmail.com

**Keywords:** vitamin D, transcription factor, Grainyhead-like, gene regulation

## Abstract

Vitamin D plays an important pleiotropic role in maintaining global homeostasis of the human body. Its functions go far beyond skeletal health, playing a crucial role in a plethora of cellular functions, as well as in extraskeletal health, ensuring the proper functioning of multiple human organs, including the skin. Genes from the Grainyhead-like (*GRHL*) family code for transcription factors necessary for the development and maintenance of various epithelia. Even though they are involved in many processes regulated by vitamin D, a direct link between vitamin D-mediated cellular pathways and *GRHL* genes has never been described. We employed various bioinformatic methods, quantitative real-time PCR, chromatin immunoprecipitation, reporter gene assays, and calcitriol treatments to investigate this issue. We report that the vitamin D receptor (VDR) binds to a regulatory region of the Grainyhead-like 1 (*GRHL1)* gene and regulates its expression. Ectopic expression of VDR and treatment with calcitriol alters the expression of the *GRHL1* gene. The evidence presented here indicates a role of VDR in the regulation of expression of *GRHL1* and correspondingly a role of *GRHL1* in mediating the actions of vitamin D.

## 1. Introduction

Vitamin D, traditionally heralded for its role in enhancing calcium absorption and maintaining serum calcium and phosphate levels for bone health, is crucial for preventing skeletal disorders such as rickets in children and osteomalacia in adults [1]. Its significance is further underscored by its role, in tandem with calcium, in staving off osteoporosis in the elderly [2]. However, the discovery of vitamin D receptors in non-skeletal tissues has shed light on its expansive role beyond bone health and calcium metabolism [3]. These findings suggest that vitamin D’s influence is far-reaching, impacting various extraskeletal functions and contributing to overall human health [4].

Vitamin D encompasses two precursor molecules: vitamin D3, or cholecalciferol, and vitamin D2, or ergocalciferol. Cholecalciferol, the primary natural source of vitamin D, is synthesized in the skin’s lower epidermis through a photochemical reaction with sunlight’s ultraviolet B rays. It can also be obtained through dietary sources like fish and egg yolks, or dietary supplements. Ergocalciferol, on the other hand, is structurally different from cholecalciferol, which influences its catabolism and results in a lower affinity for vitamin D-binding protein (VDBP). Despite these differences, both D2 and D3 undergo similar metabolic pathways and are collectively known as vitamin D. In its initial form, vitamin D is biologically inactive and requires activation through enzymatic and non-enzymatic processes. The deficiency of vitamin D has been linked to the development of autoimmune diseases such as multiple sclerosis, rheumatoid arthritis, and type 1 diabetes, underscoring its immunomodulatory role beyond bone formation and maintenance.

Skin keratinocytes, able to produce vitamin D, constitute the main source of this essential compound and possess the enzymatic machinery necessary for its conversion to the most biologically active metabolite, calcitriol (1alpha,25-dihydroxyvitamin D3), the actions of which are mediated by the nuclear receptor vitamin D receptor (VDR) [5]. VDR is a specific nuclear protein mediating the pleiotropic biological actions of calcitriol through its ability to modulate the expression of its target genes [6]. Either bound to its ligand, or in a ligand-free form, VDR regulates two central processes in the skin, basal cell proliferation and hair follicle cycling [7]. Its involvement has also been described in the context of more diverse cellular functions, such as innate immunity and formation of the permeability barrier in the upper layers of the epidermis [8]. Moreover, vitamin D synthesized in the skin upon sunlight exposure not only maintains skin barrier function but also facilitates wound healing and protects against certain dermatological conditions such as psoriasis, eczema, atopic dermatitis, and acne [9,10].

The discovery of VDR in tissues beyond bone has revolutionized our understanding of vitamin D’s effects, indicating a broader spectrum of physiological functions. VDR acts as a transcription factor, influencing gene expression by binding to vitamin D response elements (VDREs) in DNA, thereby regulating target genes [4]. To date, hundreds of genes, including dozens coding for transcription factors, have been characterized as direct VDR targets outside of the skeletal system. These include cell cycle regulatory factors such as *SP1*, *CTCF*, and *FOXO4*, genes within the cardiovascular system that regulate development, blood pressure, mitigate inflammation and enhance endothelial function, such as *GATA4* and *NF-kB*, genes that drive neurulation and nervous system function such as *POU4F2*, and within the immune and hemopoietic system, such as *BCL6*, *NFE2*, *ELF4* and *STAT3* [11,12,13]. However, the full spectrum of transcription factors regulated by VDR signaling remains to be elucidated, and targeted approaches to uncover novel synergistic pathways are necessary to further our understanding of the signaling role played by VDR during development, homeostasis and disease.

Our team has long been interested in the function of a highly conserved group of transcription factors, namely, the Grainyhead-like (GRHL) family of transcription factors. Originally identified in *Drosophila*, the vertebrate orthologues of this family comprise GRHL1, GRHL2, and GRHL3 (with the role of GRHL2 subfunctionalized into *grhl2a* and *grhl2b* in zebrafish [14]), and are highly expressed in the epidermis and epithelial layers, where they play indispensable roles in ensuring the correct epithelial structure and regeneration [15]. Beyond their involvement in skin barrier formation, GRHL1–3 factors are crucial for epithelial maintenance and regeneration of the epidermis after wounding and have been associated with skin malignancies [15]. Playing key roles in the regulation of various physiological epithelial processes, GRHL1 participates in the acquisition of the skin barrier [15], promoting epithelial integrity [16], and maintaining hair follicle homeostasis [17]. Despite these similarities that associate them with the VDR transcription factor, a direct connection between cellular pathways involving vitamin D and genes from the *GRHL1–3* family has never been reported.

Our results indicate that VDR, acting as a transcription factor, can bind to a regulatory sequence within the *GRHL1* gene promoter and regulate its expression, which implies a direct link between these two proteins.

## 2. Results

### 2.1. Bioinformatic Analyses of Potential Binding Sites for VDR in the Regulatory Regions of the GRHL1 Gene

**Figure 1:**
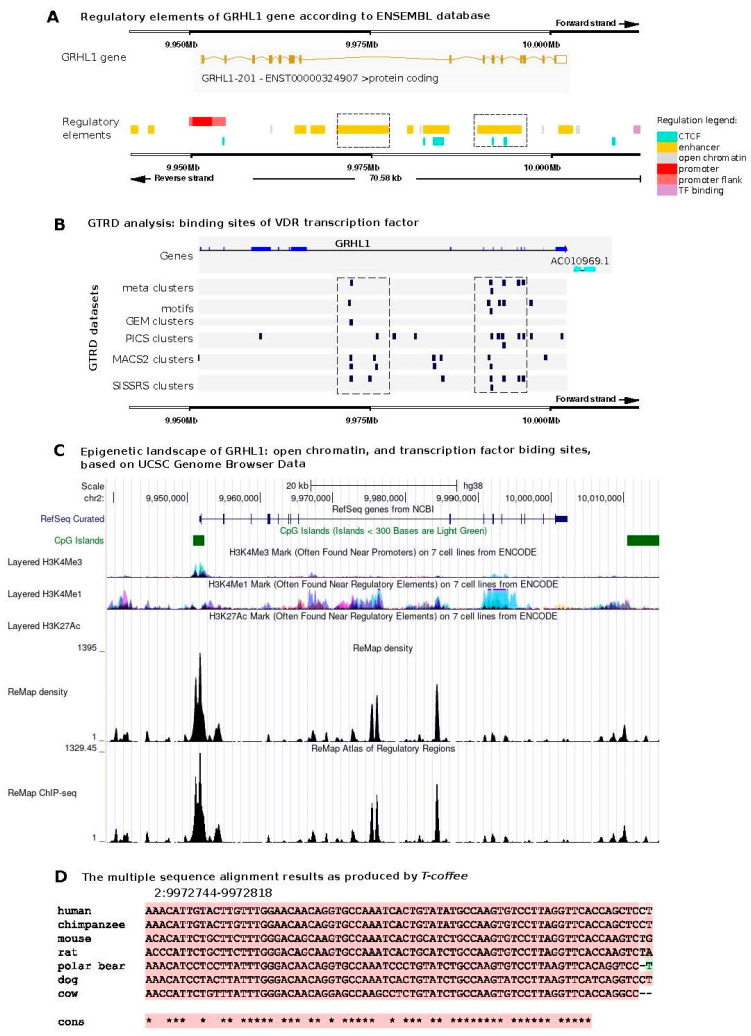
Bioinformatic predictions of transcription factor binding site (TFBS) motifs for VDR in the human *GRHL1* gene. (**A**) Genomic location of regulatory regions of the *GRHL1* gene according to the Ensembl database. (**B**) VDR binding sites identified in the *GRHL1* gene using GTRD modules: meta-clusters, motifs, and GEM–PICS–MACS2–SISSRs clusters. (**C**) Genomic location of CpG Islands, markers of open chromatin (H3K4Me1, H3K4Me3, H3K27Ac), and transcription factor binding sites (ChIP-seq). (**D**) Multiple sequence alignment of a TFBS example across different species using T-Coffee (Pro-Coffee mode). Asterisks indicate conserved nucleotides.

To identify binding sites for the VDR transcription factor within the *GRHL1* gene locus, we utilized a variety of algorithms provided by the Gene Transcription Regulation Database (GTRD), including meta clusters, motifs, and clusters derived from GEM (Genome-Wide Event Finding and Motif Discovery), PICS (Probabilistic Inference for ChIP-seq), MACS2 (Model-Based Analysis of ChIP-seq 2), and SISSRs (Site Identification from Short-Sequence Reads). Leveraging the GTRD, which houses an extensive array of uniformly processed chromatin immunoprecipitation (ChIP)-seq experimental data, facilitated the detection of VDR transcription factor binding sites. These findings, obtained through the application of diverse algorithms, showed partial overlap, and these identified VDR binding sites also matched regulatory regions within the *GRHL1* gene locus as delineated in the Ensembl database (Figure 1A,B).

Moreover, these identified sites were found to align with CpG islands and markers indicative of open chromatin states (H3K4Me1 and H3K4Me3), as documented in UCSC Genome Browser data (Figure 1C). This alignment enables a more nuanced and thorough understanding of the regulatory mechanisms that influence *GRHL1* gene expression. In addition, the comparison of the identified VDR transcription factor binding sites within the promoter region of the *GRHL1* gene across different species highlighted a significant evolutionary conservation of these regions, as depicted in Figure 1D. Such a high degree of conservation indicates the likely critical role of these regulatory elements in preserving the fundamental functions of the *GRHL1* gene across diverse organisms. The existence of these conserved VDR binding sites implies that the regulatory mechanisms governing *GRHL1* expression could be integral to its involvement in various epithelial processes.

Using the GTRD, we also identified potential VDR binding sites in the regulatory regions of the *GRHL2* and *GRHL3* genes, which are listed in Supplementary Table S1.

**Figure 2:**
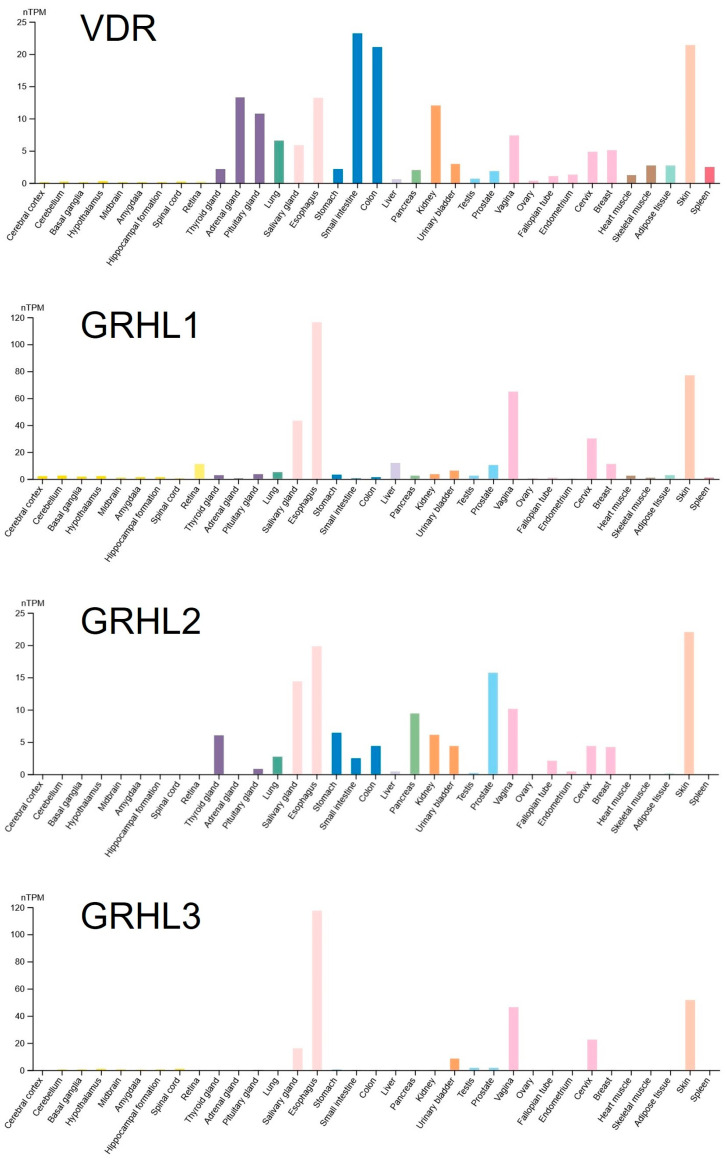
Baseline expression of *VDR* and *GRHL1–3* genes in various tissues. Based on https://www.proteinatlas.org/, last accessed 11 June 2024. TPM, transcripts per million. Database version: Genotype-Tissue Expression (GTEx) RNA-seq data v8, available from the above portal.

In parallel, we investigated in which organs *VDR* and the *GRHL1–3* genes are co-expressed. We discovered that some of their highest expression levels are found in the skin (Figure 2). For this reason, we selected for our experimental analyses the HaCaT keratinocyte cell line derived from human adult skin [18].

### 2.2. VDR and Calcitriol Regulate the Expression of the GRHL1 Gene

In order to check whether calcitriol influences the expression of *GRHL1–3* genes, HaCaT cells were transiently transfected with VDR-expressing plasmid and a control empty vector under different calcitriol treatment durations. However, in these preliminary experiments, we did not subject the cells to serum starvation, and none of the observed differences was statistically significant (Supplementary Figure S1).

**Figure 3:**
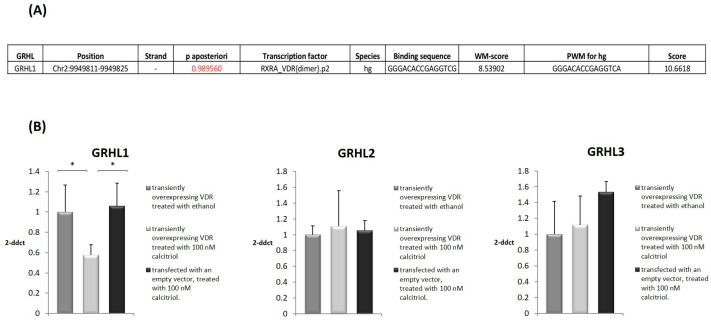
Overexpression of VDR alters mRNA level of *GRHL1* gene. (**A**) Genomic coordinates of the binding site for VDR in the promoter region of the *GRHL1* gene, obtained from the MotEvo database. (**B**) The mRNA expression levels of the *GRHL1–3* genes in HaCaT cells (1) transiently overexpressing VDR treated with ethanol, (2) transiently overexpressing VDR treated with 100 nM calcitriol, or (3) transfected with an empty vector treated with 100 nM calcitriol. The results represent relative expression of the respective target gene vs. *HPRT* genes. Data are shown as means ± SEM of experiments independently performed in triplicate, * significantly different at *p* ≤ 0.05.

In the subsequent experiment, prior to the 2 h calcitriol treatment, cells were exposed to serum starvation in serum-free DMEM overnight. Three experimental protocols were employed: (i) VDR-overexpressing cells treated with calcitriol, (ii) VDR-overexpressing cells treated with ethanol as negative control, and (iii) control vector-transfected cells treated with calcitriol. Next, using quantitative real-time PCR (qRT-PCR), we quantified the levels of expression of *GRHL1–3* genes. In VDR-overexpressing HaCaT cells treated with calcitriol, the expression of the *GRHL1* gene was downregulated by 42% when compared to the respective control cells (Figure 3B, *p* < 0.05), i.e., VDR-overexpressing cells treated with alcohol and control vector-transfected cells treated with calcitriol. We did not observe any statistically significant changes in the expression of *GRHL2* or *GRHL3* (Figure 3B). For this reason, in subsequent experiments, we focused exclusively on examining the possible regulation of expression of the *GRHL1* gene by the VDR transcription factor.

### 2.3. VDR Binds to a Regulatory Region of the GRHL1 Gene

**Figure 4:**
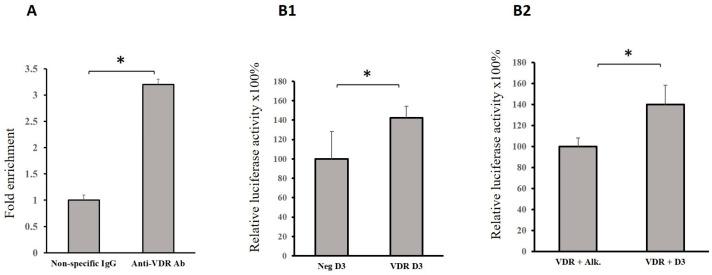
(**A**) Quantitative ChIP-PCR analysis of VDR occupancy of the *GRHL1* regulatory region was performed in HaCaT cells transfected with pcDNA3.1-K-DYK-VDR. Chromatin was immunoprecipitated with anti-DYK (FLAG) antibody or nonspecific antibody. The amount of DNA amplified from immunoprecipitated DNA was normalized to that amplified from input DNA. Data are shown as means ± SEM from experiments independently performed in triplicate, * significantly different at *p*≤ 0.05. (**B1**,**B2**) HaCaT cells were transfected with (**B1**) pcDNA3.1-K-DYK-VDR or pcDNA3.1-empty plasmid, 500 ng of the firefly luciferase vector with VDR binding site derived from the regulatory region of the *GRHL1* gene, and 25 ng pRL-CMV Renilla luciferase control reporter vector and treated with 100 nM calcitriol or (B2) pcDNA3.1-K-DYK-VDR, 500 ng of the firefly luciferase vector with VDR binding site derived from the regulatory region of the *GRHL1* gene, and 25 ng pRL-CMV Renilla luciferase control reporter vector and treated with 100 nM calcitriol or ethanol vehicle. Data are shown as means ± SEM of experiments independently performed in triplicate, * significantly different at *p*≤ 0.05.

Only one potential VDR binding site in the regulatory region of the *GRHL1* gene was identified using the MotEvo algorithm (Figure 3A). To determine whether this site is bound by VDR in living cells, ChIP analyses were carried out. Chromatin from HaCaT cells transfected with VDR-expressing vector was immunoprecipitated with 12.5 μg of anti-DYK (FLAG) antibody (ab1162, Abcam, Cambridge, UK) or 1 μg of normal rabbit IgG as negative control (10500C, Thermo Fisher Scientific, Waltham, MA, USA) and then examined by qRT-PCR using the primers listed in Table 1. As a result, we found that the investigated DNA fragments were significantly enriched in a pool precipitated with anti-DYK antibody in comparison with nonspecific IgG, indicating VDR binding to this region of the *GRHL1* promoter (Figure 4A).

We performed co-transfection studies using a fragment of the regulatory region of the *GRHL1* gene containing the putative VDR binding site fused to the reporter gene–firefly luciferase. We transiently transfected HaCaT cells with reporter constructs containing the *Luc* gene under the control of a DNA fragment with the VDR binding site from the *GRHL1* promoter and (i) VDR-overexpressing vector or (ii) a control empty vector. The cells were then treated with calcitriol for 2 h. The results obtained indicate that in cells overexpressing VDR, the expression of the *Luc* gene fused to the *GRHL1* promoter fragment was increased (Figure 4B1). In another experiment, the HaCaT cells were simultaneously transfected with the same reporter construct and a VDR-overexpressing vector and treated with either calcitriol or ethanol vehicle as negative control. The expression of the *Luc* gene fused to the *GRHL1* promoter fragment was increased in cells subjected to calcitriol treatment (Figure 4B2).

## 3. Discussion

Before the present study, there were no reports in the literature linking vitamin D signaling to the regulation of expression of the *GRHL1* gene. The novelty of our results lies in the fact that they provide the first experimental evidence of such links, and they indicate a molecular mechanism explaining these links: that the VDR transcription factor binds to the promoter of the *GRHL1* gene and regulates its expression.

VDR and GRHL1 are both transcription factors involved in the regulation of gene expression, where they play essential roles in a multitude of well-conserved cellular processes. The expression of the *GRHL1* gene is predominantly observed in various types of epithelial tissues, with the GRHL1 transcription factor being essential for structure and regeneration of various epithelia [15,16,17]. Similarly, VDR, expressed in epithelial tissues, plays a key role in the regulation of various physiological epithelial processes [19]. It has been associated with stimulation of differentiation, including permeability barrier formation, by regulating the expression levels of the tight-junction proteins claudin 2, 5, 12, and 15, strengthening the epithelial barrier function [20,21]. GRHL1 is also necessary for the functioning of skin barrier, as it regulates the expression of desmosomal cadherin desmoglein 1 (DSG1) and transglutaminase 5 (TGM5) in suprabasal layers of the epidermis and has been associated with disruption of the epidermal barrier [16,17]. These findings indicate that VDR and GRHL1 perform very similar roles in the context of skin barrier function. Despite that, there are no reports in the literature linking the functioning of VDR and GRHL1 in this or any other context. The regulation of expression of the *GRHL1* gene by the VDR transcription factor, which we describe here, may thus be relevant in the context of skin barrier function.

The interaction between these transcription factors can be crucial for maintaining epithelial integrity. The influence of VDR on gene expression profiles related to cellular junctions and barrier function can complement the role of GRHL1 in regulating genes crucial for epithelial repair and structural integrity. For example, VDR-mediated regulation of proteins such as claudin 2 and 12 enhances epithelial barrier functions, which could synergistically interact with GRHL1’s regulation of DSG1, crucial for intercellular adhesion. This presents a novel hypothesis for functional interrogation for future analyses with co-immunoprecipitation and ChIP techniques from primary mouse skin during both embryogenesis and in adults to determine potential co-regulation of these target genes by the VDR–GRHL1 pathway.

VDR is necessary to maintain hair follicle homeostasis, and its deficiency leads to alopecia, as has been shown in mouse and rat models [22,23,24]. Similarly, *Grhl1*-null mice exhibit delayed hair growth and poor hair shaft anchoring [17], which alongside the key role of DSG1 may also co-involve pathways such as Wnt/β-catenin and SHH signaling, which are crucial for hair follicle development and cycling [25,26]. Therefore, hair follicles provide yet another context in which VDR and GRHL1 perform very similar functions. The novelty of our report is that it proposes a hitherto unknown molecular mechanism of regulation of expression of the *GRHL1* gene by the VDR transcription factor, which may be relevant in the context of the functioning of hair follicles.

Both VDR and GRHL1 have been studied in the context of skin cancers. GRHL1 acts as a tumor suppressor in non-melanoma skin cancer (NMSC). *Grhl1*-null mice exposed to chemically induced skin carcinogenesis develop more squamous cell carcinomas with an earlier onset than their control wild-type littermates [27]. This phenotype is associated with aberrant terminal differentiation of keratinocytes and mild chronic skin inflammation in *Grhl1*^−/−^ mice [27]. In human NMSC, the expression of *GRHL1* is reduced, as it is negatively regulated by the oncogenic microRNA miR-21 [28]. The relationship between VDR and NMSC is more complex and not yet fully understood. Extensive evidence supports its tumor-suppressive role in this context [29]; however, the role of vitamin D in NMSC in human patients remains controversial [30,31]. Due to the contradictory evidence regarding the involvement of VDR in NMSC, it is difficult to speculate whether the regulation of expression of the *GRHL1* gene by the VDR transcription factor is relevant in this context. Nevertheless, it is possible that our findings provide a novel, hitherto unknown molecular mechanism through which VDR may act in NMSC.

The regulatory complexity of VDR and GRHL1 involves multiple layers of control, including post-translational modifications and interactions with other transcription factors and co-factors, and some studies suggest a potential synergistic or parallel regulation by VDR and GRHL1 in these processes [32]. The interactions between VDR and its co-regulators can significantly influence their transcriptional activity and the cellular response to environmental and physiological stimuli [33]. Furthermore, the modulation of VDR activity through phosphorylation by protein kinases has been noted to alter its binding efficiency and regulatory impact on target genes [34]. These interactions highlight the nuanced regulation of VDR activity, which could influence how GRHL1 functions are modulated in epithelial cells.

We are cognizant that the direction of changes in the expression of *GRHL1* and the reporter luciferase gene is not consistent: Figure 3B indicates that VDR and calcitriol negatively regulate *GRHL1* expression, while the results presented in Figure 4B suggest the opposite. We have discussed and explained such contradictions in our earlier publication [35]. Briefly, overexpression of a transcriptional transactivator can sometimes repress the transcription of its target genes: in fact, this mechanism has previously been described for the GRHL family, where GRHL3 has been shown to both induce [36] and repress [37] the expression of direct target gene E-cadherin. Also, simple rearrangements of TFBSs, such as those caused by subcloning of a promoter fragment into a reporter plasmid, can trigger qualitatively different responses to a single transcription factor. None of these contradicts our conclusion that VDR regulates the expression of the *GRHL1* gene.

The contradictions observed in the regulation of *GRHL1* by VDR, as indicated in the results of current and previous publications, suggest complex regulatory interactions that may depend heavily on the cellular context and experimental conditions [38]. These findings underline the need for further studies that employ physiologically relevant models and advanced genetic tools to dissect the intricate relationships between these transcription factors and their broader implications in epithelial biology and pathology.

Our analyses did not detect any influence of VDR on the expression of *GRHL2* or *GRHL3* in HaCaT cells, even though we identified binding sites for the VDR transcription factor in the regulatory regions of all three *GRHL1–3* genes in the GTRD (Supplementary Table S1). A possible explanation may lie in the fact that these sites were identified in different cell sets and may not be functional in keratinocytes. This hypothesis is further supported when one considers the incredible functional heterogeneity of GRHL-dependent transcriptional mechanisms. Our previously published meta-analysis of GRHL1–3 target genes highlights that the GRHL-dependent transcriptome is extremely context-specific [39], dependent on cell type, tissue of origin, developmental stage, organism and whether or not the cells were transformed, and hence it stands to reason that the upstream signaling factors that regulate GRHL1–3 function are also tightly regulated with a similar spatiotemporal profile.

Future work will focus on exploring the interaction of VDR with all three members of the GRHL1–3 family outside of keratinocyte cells, particularly during early embryogenesis in mouse and zebrafish embryos, where both VDR and GRHL1–3 factors are known to function. In particular, we aim to investigate the relationship between *grhl3* and the zebrafish orthologues of VDR (*vdra* and *vdrb*) in craniofacial development. Knockdown experiments in zebrafish revealed distinct roles for these paralogues: while loss of *vdra* has minimal effects on cartilage elements, loss of *vdrb* leads to reduced and malformed craniofacial cartilages. Simultaneous disruption of both genes results in more severe defects or complete loss of cartilage. Moreover, knockdown of *vdrb* leads to elevated expression of follistatin a (*fsta*), a bone morphogenetic protein (BMP) antagonist, in the adjacent pharyngeal endoderm [40]. As *grhl3* is expressed within the pharyngeal endoderm during zebrafish craniofacial growth [41], this spatiotemporal overlap suggests linked functions in the formation and growth of craniofacial cartilage during the establishment of the lower jaw. Additionally, we have recently shown that the murine orthologue of *Grhl2* regulates another BMP antagonist, namely, *Noggin*, during craniofacial and neural tube tissue fusion [42], leading to a further hypothesis of potential conserved functional mechanistic overlap between VDR and GRHL1–3 signaling in embryogenesis.

Many proteins that interact with VDR are already known (Table 2). However, literally nothing is known about protein partners of GRHL1 and very little about protein partners of the closely related transcription factors GRHL2 and GRHL3 [43]. Therefore, here we identify another new avenue of research to pursue. It is worth exploring whether GRHL1 interacts with any of the proteins listed in Table 2, particularly those that VDR interacts with in organs in which GRHL1 plays important functions, such as the epidermis and the kidneys [17,27,28,44,45,46]. It is even tempting to speculate that VDR, GRHL1, and some of the proteins listed in Table 2 form functional multimeric complexes. This would add yet another level of regulation of activity of the GRHL1 transcription factor and place it firmly in the context of known signaling pathways.

Studying signaling pathways involving VDR will certainly be insufficient to account for all the effects of vitamin D. It is well established that the metabolism of vitamin D is very complex: many enzymes from the cytochrome P450 superfamily are involved and many derivatives of vitamin D are produced, which perform biological functions [59,60]. Some of these derivatives act through alternative receptors, such as the aryl hydrocarbon receptor (AhR), liver X receptors (LXRs) and retinoic acid receptor-related orphan receptors (RORs) [60,61]. In future research, all these will have to be taken into account, not only in investigations aimed at deciphering the relationship between vitamin D and GRHL1–3 transcription factors but also in the studies of other functions of vitamin D.

These future investigations may take advantage of invertebrate models, in addition to the vertebrate models discussed above, as vitamin D performs important functions in all plants, animals, and even fungi [62]. In particular, insect models, such as *Drosophila melanogaster*, are proving very useful in such research [62].

## 4. Materials and Methods

### 4.1. In Silico Prediction of VDR Binding Sites in the Regulatory Elements of the GRHL1 Gene

In our previous research, we identified regulatory regions within the *GRHL1–3* genes using MotEvo, as outlined by Arnold et al. [35,63]. We then examined the predicted VDR binding sites within these regulatory regions by employing the Gene Transcription Regulation Database (GTRD) version 21.12, following standard protocols [64]. Our approach involved several techniques within the GTRD to pinpoint the VDR transcription factor binding sites in the *GRHL1* locus. These included the use of meta clusters, Genome-Wide Event Finding and Motif Discovery (GEM), Probabilistic Inference for ChIP-Seq (PICS), Model-Based Analysis of ChIP-Seq (MACS2), and Site Identification from Short-Sequence Reads (SISSRs). After identifying these binding sites, we compared them to known regulatory sequences within the *GRHL1* gene, utilizing data from the Ensembl database version 111 (https://www.ensembl.org/, last accessed 7 February 2024). This comprehensive approach allowed us to better understand the regulatory mechanisms affecting the *GRHL1* gene, particularly in relation to VDR binding. We also utilized the UCSC Genome Browser (https://genome.ucsc.edu/, last accessed 7 February 2024) to confirm whether the identified VDR binding sites in the *GRHL1* locus align with known regulatory regions, characterized by markers of open chromatin and other transcription factor binding sites.

For every *GRHL1* region pinpointed by the meta-cluster analysis as a potential VDR binding site, we retrieved its genomic alignment with corresponding sequences from six mammalian species. These species, selected from Ensembl, include the chimpanzee (*Pan troglodytes*), mouse (*Mus musculus*), rat (*Rattus norvegicus*), polar bear (*Ursus maritimus*), dog (*Canis lupus*), and cow (*Bos taurus*). We conducted these alignments through the “Comparative genomics” feature in the Ensembl Genome Browser, focusing on the human genomic coordinates for these regions. The alignments were executed using the BLASTz/LASTz algorithms, with the results saved in the Fasta file format for further analysis. Multiple alignments were carried out with T-Coffee (ver. 11.0) [65] to determine if the VDR binding sites are conserved in other organisms. We used Pro-Coffee mode. The command line used to execute the alignment was: t_coffee -in=data_411e99a0.in -mode=procoffee -output=score_html clustalw_aln fasta_aln score_ascii phylip -maxnseq=150 -maxlen=10000 -case=upper -seqnos=off -outorder=input -run_name=result -multi_core=4 -quiet=stdout.

### 4.2. Cell Culture

Human immortalized keratinocyte HaCaT cells [18] were cultured in DMEM GlutaMAX medium supplemented with 10% fetal bovine serum and 100 IU/mL penicillin–streptomycin at 37 °C in a humidified incubator under 5% CO_2_. Cell culture reagents were purchased from Thermo Fisher Scientific.

### 4.3. Plasmids

The pcDNA3.1-K-DYK-VDR plasmid was purchased from GenScript and the control pcDNA3.1 plasmid was purchased from Invitrogen. Primers flanking a regulatory element of the *GRHL1* gene including a VDR binding site were used to PCR amplify this element and clone it into the firefly luciferase vector with SV40 promoter (pGL3-promoter) (Promega, Madison, WA, USA) using In-Fusion^®^ HD Cloning Kit (Takara Bio, Kusatsu, Shiga Prefecture, Japan), according to a protocol provided by the manufacturer. The construct was verified by sequencing. The control vector with the Renilla luciferase gene (pRL-CMV) was also purchased from Promega. Primers used for cloning are listed in Table 3.

### 4.4. Total RNA Extraction, Reverse Transcription, and qRT-PCR Assays

We grew HaCaT cells in 6-well plates with a well diameter of 34.8 mm. In each of the experimental procedures described in Section 4.4, Section 4.5 and Section 4.6, we followed the protocols supplied by the manufacturers. For cell transfections, we used Lipofectamine 3000 (Thermo Fisher Scientific). In each transfection, we used 1.0 μg of pcDNA3.1-K-DYK-VDR or pcDNA3.1-empty plasmids (negative control). After transfections, we cultured the cells for 24 h. Subsequently, we extracted and purified total RNA using the Total RNA Mini Plus Kit (A&A Biotechnology). We quantified the yield of RNA by measuring the absorbance at 260 nm, and we evaluated RNA purity according to the A260/A280 and A260/A230 ratio (NanoDrop ND-1000, Thermo Fisher Scientific). We then examined our samples by electrophoresis on 1.5% agarose, where the presence of sharp bands corresponding to 18S and 28S rRNA confirmed the integrity of total RNA. Subsequently we reverse-transcribed 1 μg of total RNA into first-strand cDNA with ReadyScript cDNA Synthesis Mix (Sigma-Aldrich). We carried out qRT-PCR in triplicate on a StepOnePlus Real Time PCR system (Applied Biosystems), using TaqMan Fast Universal PCR Master Mix No AmpErase UNG (Thermo Fisher Scientific) and TaqMan probes (Thermo Fisher Scientific). We normalized gene expression levels to the hypoxanthine phosphoribosyltransferase 1 housekeeping gene (*HPRT1*) (the highest stability based on the literature [66]) and calculated using the 2^−ΔΔCt^ method [67]. In our experiments, we used the following TaqMan probes: Hs01119372_m1 for *GRHL1*, Hs00227745_m1 for *GRHL2*, Hs00297962_m1 for *GRHL3*, and Hs02800695_m1 for *HPRT1*. We determined statistical differences for relative expression levels using Student’s *t*-test. We considered *p* ≤ 0.05 to be statistically significant.

### 4.5. Chromatin Immunoprecipitation Assays (ChIP)

We cultured and transfected HaCaT cells as described in Section 4.4 (above). For transfections, we used 1.0 μg of pcDNA3.1-K-DYK-VDR plasmid. After transfections, we cultured the cells for 24 h. Subsequently, we cross-linked the cells for 10 min by adding formaldehyde to the final concentration of 1%. We sonicated the cross-linked material for 25 min (30 s on/30 s off) (Bioruptor, Diagenode) to generate ~500 bp DNA fragments. We isolated chromatin using an Imprint Chromatin Immunoprecipitation Kit (Sigma-Aldrich). We immunoprecipitated DNA fragments with 12.5 μg of anti-DYK (FLAG) antibody (ab1162, Abcam) and 1 μg of normal rabbit IgG as negative control (10500C, Thermo Fisher Scientific), and analyzed them in triplicate using qRT-PCR. We calculated fold changes related to 10% input delta Ct as 2^−ΔΔCt^ [67]. Primers used for ChIP are listed in Table 1. We determined statistical differences for relative expression levels using Student’s *t*-test. We considered *p* ≤ 0.05 to be statistically significant.

### 4.6. Reporter Gene Assays

We cultured and transfected HaCaT cells as described in Section 4.4 (above). For transfections, we used 500 ng of pcDNA3.1-DYK-VDR or pcDNA3.1-empty plasmid, 25 ng pRL-CMV and 500 ng of the firefly luciferase vector with VDR binding site (described in Section 4.3—Plasmids). After transfections, we cultured the cells for 24 h. Subsequently, we lysed the cells using a lysis buffer provided in the Dual-Luciferase Reporter Assay System (Promega), and we measured the luciferase activity using materials supplied in this system and a Tecan Infinite M1000 PRO luminometer. We calculated and normalized relative reporter activity based on Renilla luciferase activity. We carried out all assays in triplicate twice. We performed statistical evaluations using Student’s *t*-test. We considered *p* ≤ 0.05 to be statistically significant.

### 4.7. Calcitriol Treatment

The cells were exposed to serum starvation in serum-free DMEM for 12 h and treated with calcitriol dissolved in ethanol (100 nM final culture concentration) (Sigma) or ethanol vehicle as negative control for 2 h prior to the experiments.

## 5. Conclusions

In this study, we identified a potential VDR binding site in the promoter region of the *GRHL1* gene. We experimentally confirmed that VDR binds to this site in a human keratinocyte cell line. We demonstrated that VDR and calcitriol regulate the expression of the *GRHL1* gene. Despite previously known numerous parallels between vitamin D signaling and signaling involving the GRHL1–3 factors, a direct connection between these two cellular pathways has not been reported before. Our findings indicate such a connection. Thus, we describe a hitherto unknown potential molecular mechanism through which vitamin D can act, as well as a novel mechanism of regulation of expression of the *GRHL1* gene.

## Figures and Tables

**Table 1:** List of ChIP qRT-PCR primers.

Binding Site	Primer	Sequence (5′→3′)
VDR in *GRHL1* promoter	Forward	GGGCACAGAGGAGGGACT
	Reverse	GAGACAGAAGACGGGGACAC

**Table 2:** Vitamin D receptor-mediated transcription factor regulation across organ systems.

Transcription Factor	Organism	Tissue/Organ/Cell Type	Details of Interaction	Source
Liver				
RXR	Human, mouse, rat	Liver	Forms heterodimers with VDR to regulate gene transcription.	[47]
TFIIB	Human, mouse	Liver	Interacts directly with VDR to facilitate transcription initiation.	[47]
p300/CBP	Human, mouse, rat	Liver	Acts as a histone acetyltransferase, enhancing transcription by relaxing DNA.	[48]
HNF-4α	Human, mouse, rat	Liver	Interacts with VDR to regulate metabolic processes in liver cells.	[48]
Kidney				
RXR	Human, mouse, rat	Kidney	Forms heterodimers with VDR to regulate gene transcription.	[47]
TFIIB	Human, mouse	Kidney	Interacts directly with VDR to facilitate transcription initiation.	[47]
p300/CBP	Human, mouse, rat	Kidney	Acts as a histone acetyltransferase, enhancing transcription by relaxing DNA.	[48]
HNF-4α	Human, mouse, rat	Kidney	Interacts with VDR to regulate metabolic processes in kidney cells.	[48]
Intestine				
RXR	Human, mouse, rat	Intestine	Forms heterodimers with VDR to regulate gene transcription.	[47]
TFIIB	Human, mouse	Intestine	Interacts directly with VDR to facilitate transcription initiation.	[47]
p300/CBP	Human, rat, mouse	Intestine	Acts as a histone acetyltransferase, enhancing transcription by relaxing DNA.	[48]
HNF-4α	Human, mouse, rat	Intestine	Interacts with VDR to regulate metabolic processes in intestinal cells.	[48]
Bone				
RXR	Human, mouse, rat	Osteoblasts, osteoclasts	Forms heterodimers with VDR to regulate gene transcription.	[47]
NCoR	Human, mouse	Osteoblasts, osteoclasts	Acts as a corepressor that interacts with VDR to suppress transcription.	[49]
SMRT	Human, mouse	Osteoblasts, osteoclasts	Another corepressor that interacts with VDR to regulate gene expression.	[49]
SRC-1	Human, mouse	Osteoblasts, osteoclasts	Coactivator that enhances VDR-mediated transcription.	[50]
Muscle Cells				
MyoD	Human, mouse	Myoblasts	Interacts with VDR to influence muscle cell differentiation and growth.	[49]
Skin Cells				
TIF2	Human, mouse	Keratinocytes	Coactivator that interacts with VDR to enhance transcription in skin cells.	[51]
Nervous System				
NF-1	Human, mouse	Neurons, glial cells	Interacts with VDR to regulate genes involved in nervous system development and function.	[32]
FOXO3	Human, mouse	Neurons, glial cells	Interacts with VDR to regulate genes involved in stress resistance, metabolism, and neuronal function.	[32]
Cancer Cells				
c-Myc	Human, mouse	Various cancer cells (colon, breast, prostate)	Oncogene that interacts with VDR, influencing cell proliferation and cancer progression.	[32]
HOXB13	Human, mouse	Prostate cancer cells	Interacts with VDR in prostate cancer cells to regulate gene expression related to cancer progression.	[52]
Immune System				
NF-κB	Human, mouse, rat	Immune cells (T cells, B cells)	Modulates immune responses and inflammation through interaction with VDR.	[32]
AP-1	Human, mouse, rat	Immune cells (T cells, B cells)	Regulates gene expression in response to a variety of stimuli, interacting with VDR to modulate immune functions.	[32]
Alien	Human, mouse	Immune cells (T cells, B cells)	Acts as a corepressor that interacts with VDR to modulate transcriptional repression.	[53]
BCL6	Human, mouse	B cells, T cells	Regulates differentiation and function of T cells and B cells, interacting with VDR.	[32]
STAT3	Human, mouse	Various immune cells (T cells, NK cells, macrophages)	Involved in the signaling pathways that mediate immune responses and interacts with VDR.	[32]
GATA3	Human, mouse	T cells, Th2 cells	Interacts with VDR to regulate immune responses, particularly in T-helper 2 (Th2) cells.	[32]
NFAT	Human, mouse	Immune cells (T cells, B cells)	Interacts with VDR to modulate the expression of immune-related genes.	[32]
IRF1	Human, mouse	Immune cells (T cells, B cells)	Interacts with VDR to regulate the transcription of interferon-responsive genes.	[32]
c-Fos	Human, mouse	Immune cells (T cells, B cells)	Component of the AP-1 transcription factor, interacts with VDR to modulate immune responses.	[32]
Development				
Oct4	Human, mouse	Embryonic stem cells	Interacts with VDR to regulate gene expression during development.	[54]
Wnt Signaling				
TCF/LEF	Human, mouse, rat	Various cells (liver, kidney, intestine, bone)	Mediates gene expression changes in response to Wnt signaling, interacts with VDR.	[48]
TCF7L2	Human	Various tissues (liver, kidney, intestine, bone)	Interacts with VDR in the Wnt signaling pathway to regulate gene expression.	[55]
Transcription Regulation				
GRIP1	Human, mouse	Various cells (liver, kidney, intestine, bone)	Coactivator that interacts with VDR to enhance transcriptional activation.	[54]
RAC3	Human, mouse	Various cells (liver, kidney, intestine, bone)	Coactivator that works with VDR to enhance transcription.	[54]
SKIP	Human, mouse	Various cells (liver, kidney, intestine, bone)	Involved in the assembly of the transcriptional complex with VDR.	[56]
VDRE Binding Proteins	Human	Various cells (liver, kidney, intestine, bone)	Proteins that bind to Vitamin D Response Elements (VDREs) to regulate gene expression in coordination with VDR.	[57]
General				
TRAM-1	Human, mouse, rat	Various tissues (liver, kidney, intestine, bone)	Coactivator that enhances VDR-mediated transcription.	[50]
TIF1	Human, mouse	Various tissues (liver, kidney, intestine, bone)	Interacts with VDR to modulate transcription.	[51]
TRAP220	Human, mouse, rat	Various tissues (liver, kidney, intestine, bone)	Part of the mediator complex, linking transcriptional regulators to the RNA polymerase II initiation complex.	[54]
SUG1	Human, mouse	Various tissues (liver, kidney, intestine, bone)	Component of the 26S proteasome, involved in non-proteolytic roles like nuclear receptor-mediated transcription.	[54]
BAF60a	Human, mouse	Various tissues (liver, kidney, intestine, bone)	Part of the SWI/SNF chromatin remodeling complex, facilitates transcriptional activation with VDR.	[54]
NCoA1	Human, mouse	Various tissues (liver, kidney, intestine, bone)	Enhances the transcriptional activities of steroid hormone receptors like VDR.	[51]
c-Jun	Human, mouse, rat	Various tissues (liver, kidney, intestine, bone)	Component of the AP-1 transcription factor, interacts with VDR to respond to stress signals and inflammation.	[32]
CREB	Human, mouse	Various tissues (liver, kidney, intestine, bone)	Interacts with VDR to mediate cAMP response element-binding protein transcriptional activities.	[48]
COUP-TF	Human, mouse	Various tissues (liver, kidney, intestine, bone)	Interacts with VDR to regulate gene expression across various tissues.	[52]
USF1	Human, mouse	Various tissues (liver, kidney, intestine, bone)	Interacts with VDR to regulate gene expression across various tissues.	[52]
GFI1	Human, mouse	Various tissues (liver, kidney, intestine, bone)	Interacts with VDR to regulate gene expression across various tissues.	[52]
SUG2	Human, mouse	Various tissues (liver, kidney, intestine, bone)	Component of the 26S proteasome, involved in nuclear receptor-mediated transcription with VDR.	[54]
VDR-AP (alternative pocket)	Human, mouse	Various tissues (liver, kidney, intestine, bone)	Represents a different binding conformation within VDR that might interact uniquely with ligands or coregulators.	[58]

**Table 3:** List of oligonucleotides used for cloning.

Direction	Sequences of Oligonucleotides (5′→3′)
Forward	actGAGCTCcaaacaacaggctgcatgga
Reverse	actgCTCGAGcgagagtctgttttggacgt

## Data Availability

All data generated or analyzed during this study are included in the manuscript and/or the Supplementary Materials.

## Supplementary Materials

The following supporting information can be downloaded at https://www.mdpi.com/article/10.3390/ijms25147913/s1.
