# Supplementary material for: Vitamin D Receptor Regulates the Expression of the Grainyhead-Like 1 Gene
Source: Int J Mol Sci. 2024 Jul 19;25(14):7913. doi: 10.3390/ijms25147913 (PMC11276664; doi:10.3390/ijms25147913)
Supplement: Supplementary file 1 [file ijms-25-07913-s001.zip › Supplementary Figure S1.pdf]

(A)

(1)

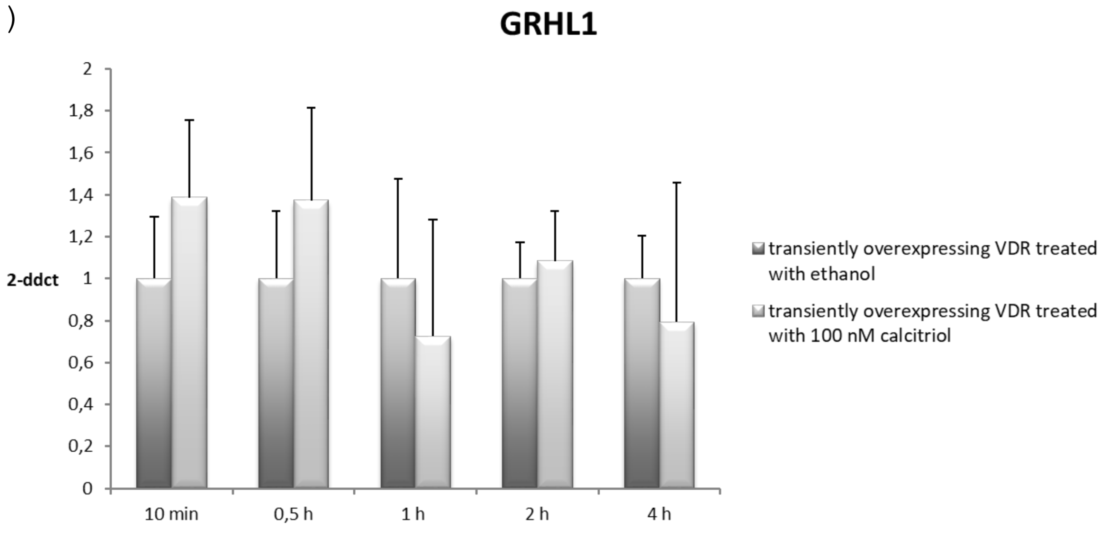
GRHL1
2-ddct
0
0,2
0,4
0,6
0,8
1
1,2
1,4
1,6
1,8
2
10 min
0,5 h
1 h
2 h
4 h
transiently overexpressing VDR treated with ethanol
transiently overexpressing VDR treated with 100 nM calcitriol

(2)

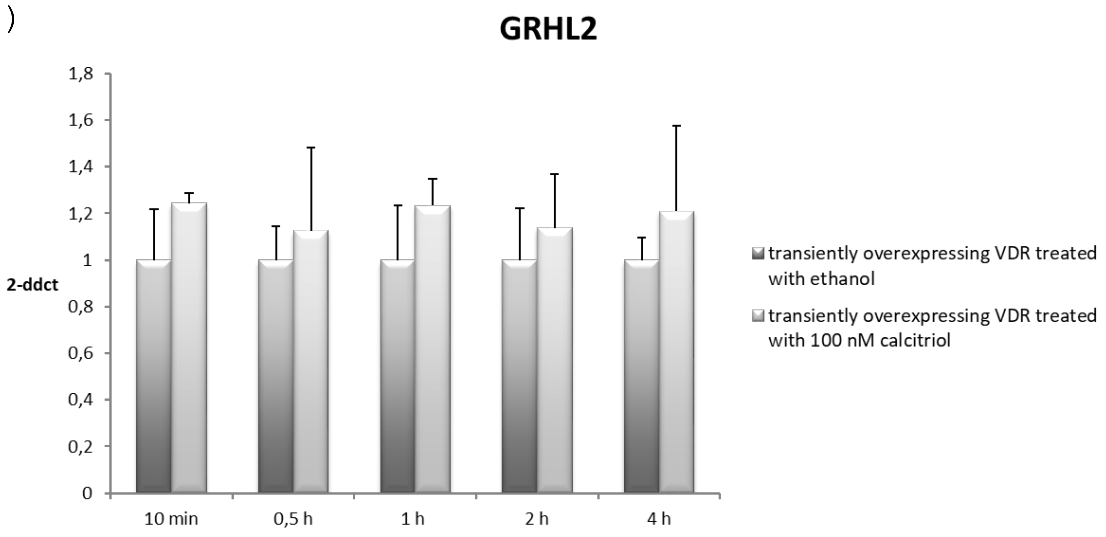
GRHL2
2-ddct
0
0,2
0,4
0,6
0,8
1
1,2
1,4
1,6
1,8
10 min
0,5 h
1 h
2 h
4 h
transiently overexpressing VDR treated with ethanol
transiently overexpressing VDR treated with 100 nM calcitriol

(3)

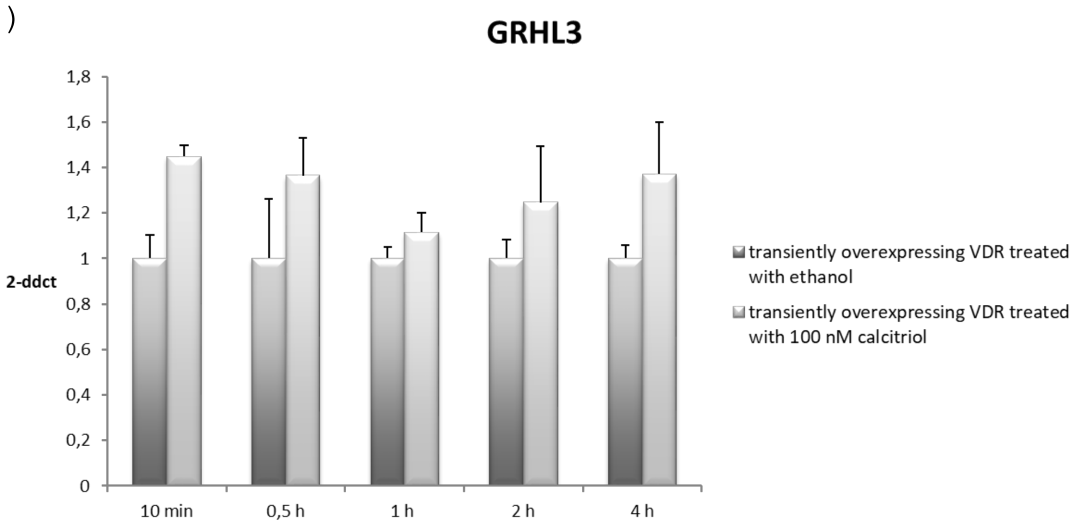
GRHL3
2-ddct
0
0,2
0,4
0,6
0,8
1
1,2
1,4
1,6
1,8
10 min
0,5 h
1 h
2 h
4 h
transiently overexpressing VDR treated with ethanol
transiently overexpressing VDR treated with 100 nM calcitriol

(B)

(1)

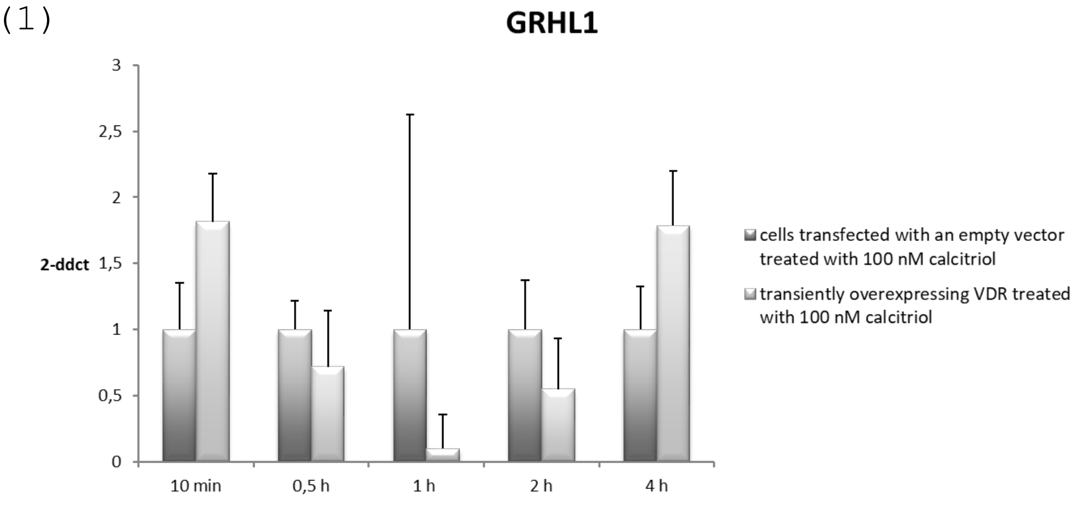
GRHL1
3
2,5
2
1,5
1
0,5
0
2-ddct
10 min
0,5 h
1 h
2 h
4 h
cells transfected with an empty vector treated with 100 nM calcitriol
transiently overexpressing VDR treated with 100 nM calcitriol

(2)

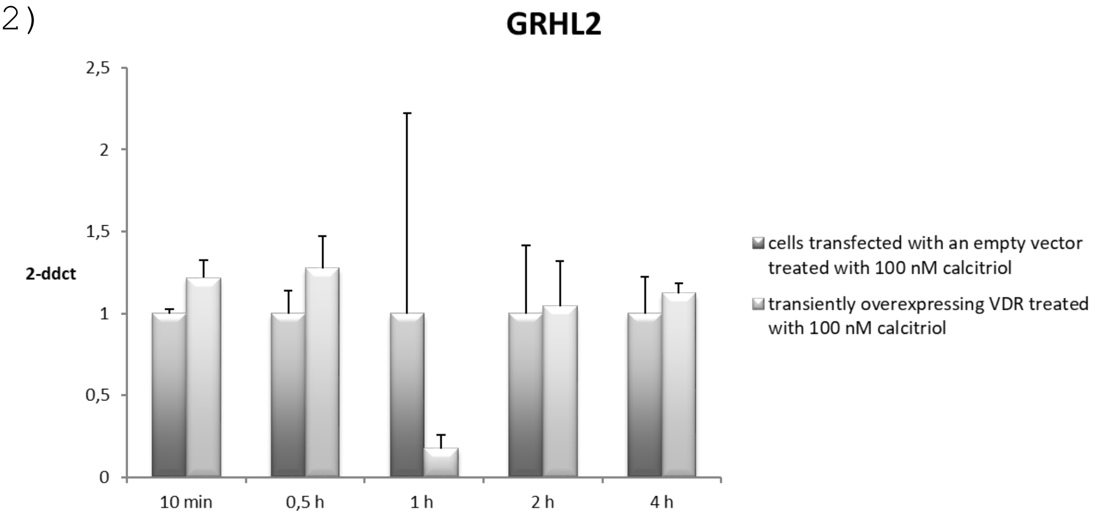
GRHL2
2,5
2
1,5
1
0,5
0
2-ddct
10 min
0,5 h
1 h
2 h
4 h
cells transfected with an empty vector treated with 100 nM calcitriol
transiently overexpressing VDR treated with 100 nM calcitriol

(3)

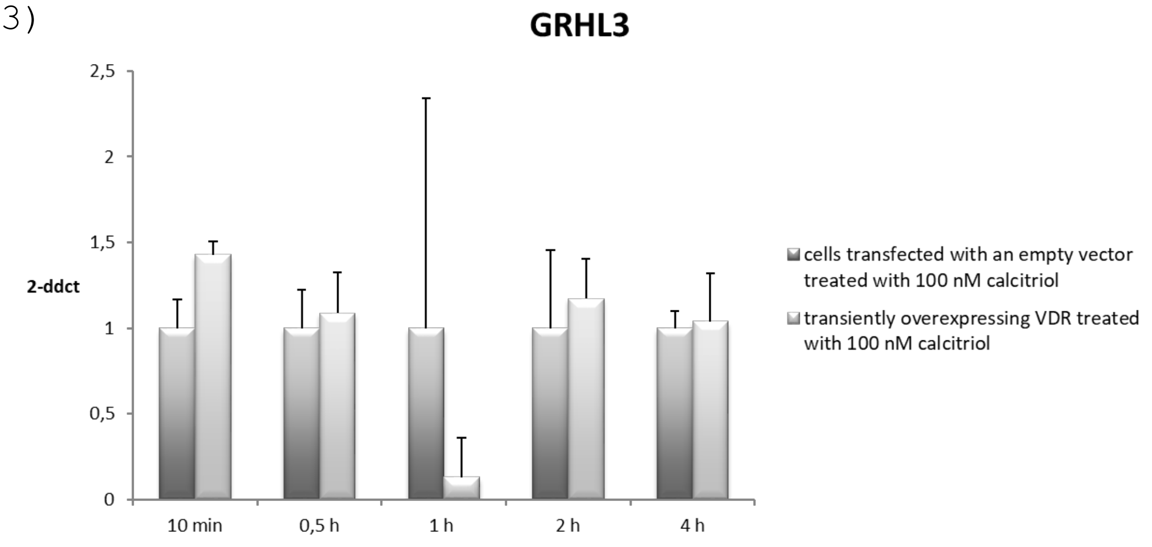
GRHL3
2,5
2
1,5
1
0,5
0
2-ddct
10 min
0,5 h
1 h
2 h
4 h
cells transfected with an empty vector treated with 100 nM calcitriol
transiently overexpressing VDR treated with 100 nM calcitriol

(C)

(1)

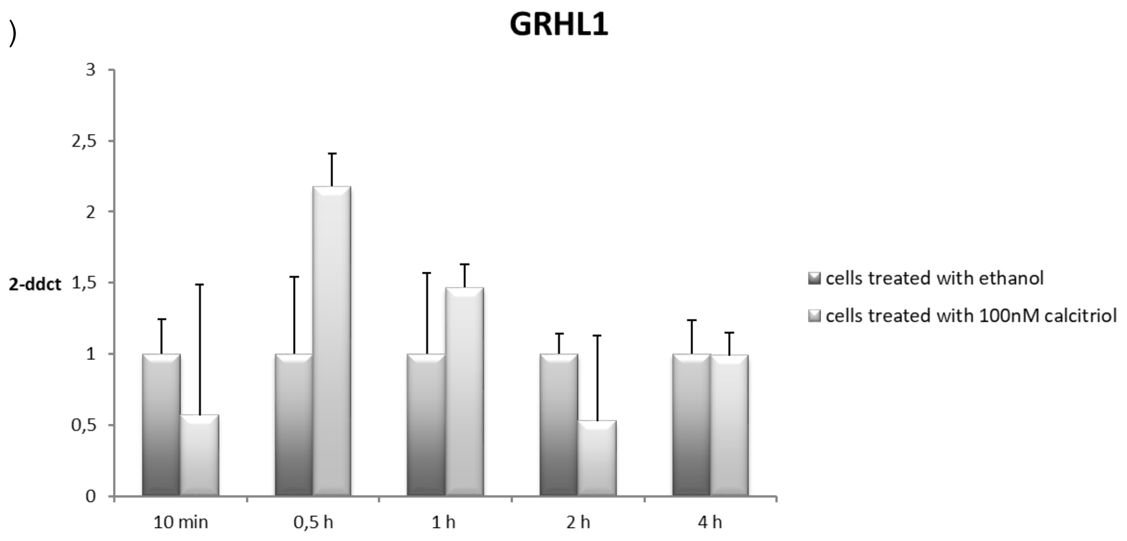
GRHL1
2-ddct
3
2,5
2
1,5
1
0,5
0
10 min
0,5 h
1 h
2 h
4 h
cells treated with ethanol
cells treated with 100nM calcitriol

(2)

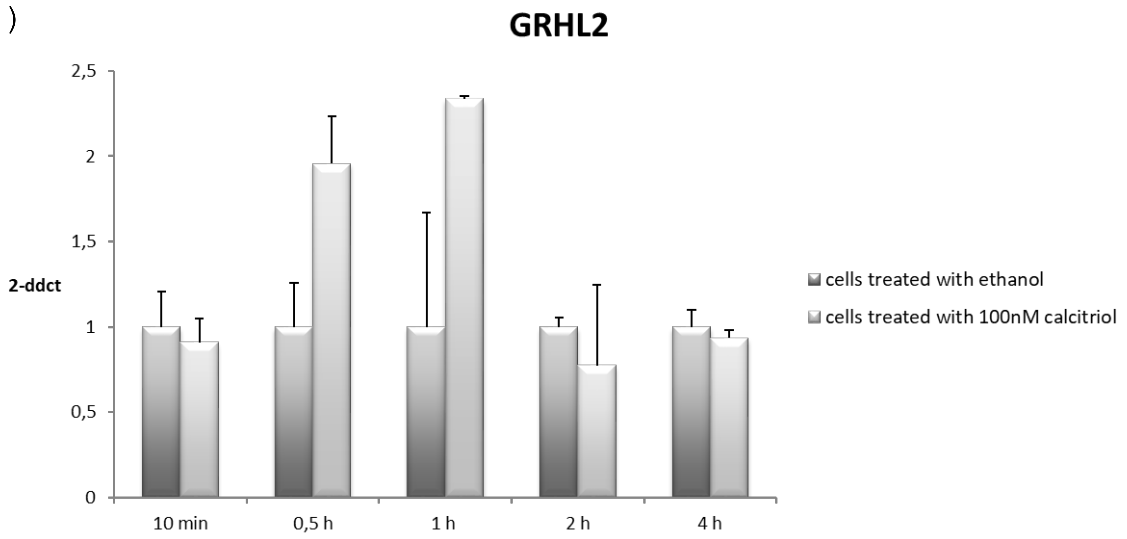
GRHL2
2-ddct
2,5
2
1,5
1
0,5
0
10 min
0,5 h
1 h
2 h
4 h
cells treated with ethanol
cells treated with 100nM calcitriol

(3)

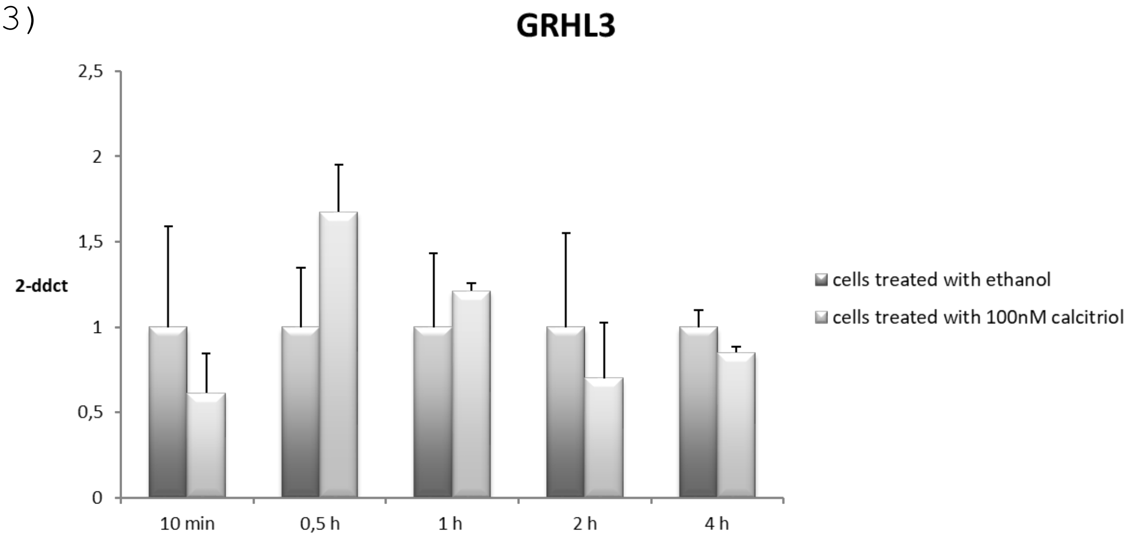
GRHL3
2-ddct
2,5
2
1,5
1
0,5
0
10 min
0,5 h
1 h
2 h
4 h
cells treated with ethanol
cells treated with 100nM calcitriol

Supplementary Figure S1. The mRNA expression levels of GRHL1-3 genes in HaCaT cells.

(A) HaCaT cells transfected with the pcDNA3.1-VDR, treated with 100 nM calcitriol or ethanol vehicle (control group) for 10 min, 30 min, 1 hour, 2 hours, or 4 hours (B) HaCaT cells transfected with the pcDNA3.1-VDR-DYK vector treated with 100 nM calcitriol for (1) for 10 min, 30 min, 1 hour, 2 hours, or 4 hours (C) HaCaT cells treated with 100 nM calcitriol or ethanol (control group) for for 10 min, 30 min, 1 hour, 2 hours, or 4 hours. The results represent relative expression of the respective target gene vs HPRT gene. Data are shown as means ± SD of experiments independently performed in triplicate
